# Artificial intelligence usage, breakthrough innovation, and innovation performance in high-tech enterprises: the nonlinear moderating role of Not-Invented-Here Syndrome

**DOI:** 10.3389/frai.2025.1699860

**Published:** 2026-01-30

**Authors:** Dian Guan, Zexia Wang, Wucheng Han, Yinqiang Pei

**Affiliations:** 1 Xihua University, Chengdu, China; 2 University of Electronic Science and Technology of China, Chengdu, China; 3 Western China Transformation Center for Advanced Technological Achievements, Chengdu, China

**Keywords:** AI use, breakthrough innovation, innovation performance, NIHS, nonlinear moderation

## Abstract

The integration of artificial intelligence (AI) with frontier technologies such as large language models and quantum computing has significantly enhanced enterprises’ potential for breakthrough innovation, becoming a critical driver of innovation performance. However, the internal mechanisms and boundary conditions through which AI influences innovation performance via breakthrough innovation remain unclear, requiring further exploration to deepen our understanding of AI’s crucial role in organizational innovation. Drawing on the resource-based view (RBV), this study systematically investigates the impact of AI use on innovation performance, emphasizing the mediating role of breakthrough innovation and the moderating effect of Not-Invented-Here Syndrome (NIHS). Data were collected from 355 global high-tech enterprises via the Prolific platform and analyzed using partial least squares structural equation modeling (PLS-SEM). The findings demonstrate that AI use positively impacts innovation performance, with breakthrough innovation serving as a significant mediator. NIHS exhibits an inverted U-shaped moderating effect on the relationship between AI use and innovation performance, while displaying a U-shaped moderating effect between breakthrough innovation and innovation performance. This study provides initial empirical evidence that AI promotes breakthrough innovation in high-tech enterprises, thus enhancing innovation performance, unveiling the ‘black box’ of how AI influences innovation performance through breakthrough innovation. Moreover, it explores the nonlinear moderating role of NIHS, reinforcing the applicability of RBV in the digital era. This research also offers practical guidance for high-tech enterprises to optimize resource allocation and implement breakthrough innovation strategies in AI-driven innovation environments to achieve superior innovation performance and competitive advantage.

## 1 Introduction

Rapid advances in artificial intelligence (AI) are reconfiguring enterprises’ innovation logics and sources of competitive advantage (Haenlein and Kaplan, 2019; Krakowski et al., 2023). Enterprises are allocating growing resources to AI to streamline operations, enhance decision quality, and develop novel products and services. Industry evidence suggests broad diffusion across manufacturing, healthcare, financial services, and retail, positioning AI as a distinctive resource for improving innovation performance and competitive positioning. The rise of generative AI—underpinned by foundation models, machine learning, natural language processing, and, prospectively, quantum computing—has further expanded the frontier for breakthrough innovation (Balasubramanian et al., 2022; Mariani and Dwivedi, 2024). Yet these opportunities are tempered by significant implementation challenges: sizable capital outlays for infrastructure, constrained access to high-quality data, shortages of talent that integrate technical and domain expertise, organizational resistance to change, and persistent concerns over privacy and algorithmic bias (Grewal et al., 2021). Returns are also heterogeneous across industries and use cases; while some enterprises realize material gains, others fall short of expectations, and in some instances, initiatives are discontinued due to insufficient value creation (Felten et al., 2021). These patterns underscore the need to understand how enterprises can leverage AI effectively to enhance innovation performance.

Within the resource-based view (RBV), AI is increasingly conceptualized as a strategic resource that can enable sustained advantage and superior innovation outcomes (Krakowski et al., 2023; Mariani et al., 2023b). Beyond a technical artifact, AI operates through data, algorithms, and complementary organizational assets to support information processing, resource generation, and decision making, thereby facilitating dynamic resource orchestration (Raisch and Krakowski, 2021). Prior research indicates that AI can strengthen the identification, acquisition, and allocation of innovation-relevant resources, reshape search and selection processes, and ultimately elevate innovation outcomes (Mariani et al., 2023a; Roberts and Candi, 2024). At the same time, AI adoption introduces novel innovation paradigms that may disrupt established routines and resource deployment strategies. Factors such as top management’s change orientation, technological opacity and bias, organizational restructuring, and the magnitude of financial and technical commitments complicate the performance implications (Arias-P’Erez and V’Elez-Jaramillo, 2022; Han et al., 2025). Consequently, rigorous examination of AI’s real-world effectiveness has become a research priority (Du and Xie, 2021; Grewal et al., 2021). Clarifying the specific pathways, mechanisms, and boundary conditions through which AI contributes to innovation performance is therefore an essential agenda for further inquiry.

AI, as a frontier technology transcending human cognitive limitations, serves as crucial support for breakthrough innovation due to its robust data processing, pattern generation, and resource emergence (Balasubramanian et al., 2022; Cooper et al., 2023). Breakthrough innovation refers to creating novel technologies, products, or services previously absent from the market, triggering substantial market changes and reshaping industry standards and development patterns (Capponi et al., 2022; Datta and Srivastava, 2023). Conventional perspectives suggest that AI can only recombine existing knowledge and resources; however, recent technological advances enable AI to autonomously generate and emergently produce new knowledge and capture complex system dynamics. This capacity allows firms to break free from innovation path dependence based on existing resources and knowledge (Mariani and Dwivedi, 2024; Roberts and Candi, 2024), facilitating entirely new technological combinations and application scenarios, thus driving breakthrough innovation and ultimately enhancing innovation performance (Silva et al., 2017). Yet, the mediating mechanism of breakthrough innovation in AI’s impact on innovation performance remains unclear and requires deeper investigation.

In translating AI-driven breakthrough innovation into superior innovation performance, the cognitive and decision-making attributes of top management teams are pivotal (Wilms et al., 2019; Han et al., 2025), particularly through their openness to external knowledge and resources. Not-Invented-Here Syndrome (NIHS)—an orientation of reservation or resistance toward external innovation inputs (Hannen et al., 2019; Amann et al., 2021)—conditions how AI use influences performance outcomes. Extant findings on NIHS and enterprise innovation are mixed. Some studies argue that NIHS can strengthen internal resource integration and architectural coherence, thereby enhancing innovation performance (Felin and Zenger, 2014; Manzini et al., 2017). Others show that excessive NIHS induces cognitive rigidity, suppresses the variety of novel inputs, and ultimately impedes breakthrough innovation (Ismail et al., 2023). These inconsistencies point to nonlinear effects. Psychologically, a moderate level of NIHS may balance identity protection with learning openness, whereas extreme levels on either side disrupt this equilibrium (Antons and Piller, 2015; Witthöft et al., 2025). Organizationally, NIHS creates tension between external knowledge absorption and capability protection, plausibly yielding curvilinear (e.g., inverted U-shaped) relationships with innovation performance (Hannen et al., 2019). Building on this logic, we conceptualize NIHS as a multilevel construct and theorize a nonlinear moderating role of NIHS in the relationship between AI-driven breakthrough innovation and enterprise innovation performance, thereby motivating systematic inquiry into the underlying mechanisms and boundary conditions.

Based on the above analysis, this study addresses the following research questions: (1) Can AI enhance firms’ innovation performance by promoting breakthrough innovation? (2) What role does NIHS play in the relationships among AI use, breakthrough innovation, and innovation performance? To answer these questions, we conducted a two-stage online survey through the Prolific platform, collecting data from 355 high-tech enterprises globally. High-tech firms, characterized by technology-intensive innovation orientation, robust data assets, advanced R&D, and agile organizational structures, provide an ideal context for studying AI-enabled breakthrough innovation. Data analysis was conducted using PLS-SEM to test the research hypotheses.

This research contributes significantly to the literature on AI and innovation management. First, it systematically elucidates the mediating mechanism of breakthrough innovation in the relationship between AI use and innovation performance, deepening the understanding of the complex processes through which AI influences innovation performance (Du and Xie, 2021; Rammer et al., 2022). Second, it examines the nonlinear moderating role and mechanisms of NIHS as a boundary condition, challenging the conventional view of NIHS’s linear effects (Ismail et al., 2023). Third, it enriches and extends the RBV framework in the digital era, emphasizing AI’s strategic importance for organizational competitive advantage and innovation performance (Helfat et al., 2023).

The remainder of this paper is structured as follows. Section 2 reviews theoretical backgrounds and formulates hypotheses. Section 3 outlines the methodology, including sampling, data collection, and analytical techniques. Section 4 presents results from empirical analysis. Section 5 discusses findings, implications, limitations, and future research directions. Section 6 concludes the paper.

## 2 Theoretical background and hypotheses development

### 2.1 Resource-based view

The resource-based view (RBV) offers a coherent theoretical lens for this study, guiding a systematic analysis of how enterprises deploy strategic resources to secure competitive advantage and elevate innovation performance (Barney, 2001). Under RBV, resources that are valuable, rare, inimitable, and non-substitutable constitute the foundations of sustained competitiveness and serve as engines of innovation (Terziovski, 2010; Barney et al., 2021). Such resources span tangible assets—advanced equipment, financial capital, and physical infrastructure—and intangible assets—brand reputation, intellectual property, and employee competencies (Jancenelle, 2021). Through effective integration and orchestration of these heterogeneous resources, enterprises develop pioneering technologies, products, and services, thereby strengthening innovative outputs and overall innovation performance.

Against the backdrop of rapid digitization, artificial intelligence (AI) has emerged as a pivotal strategic resource within the RBV paradigm. Recent scholarship extends RBV to encompass AI-enabled resources, enriching its theoretical scope and contemporary relevance (Helfat et al., 2023). As a novel class of strategic resource, AI transcends traditional asset constraints by introducing digital and data-intensive capabilities that reconfigure enterprises’ innovation processes (Sullivan and Wamba, 2024). Its technological affordances reshape how resources are accessed, combined, and deployed, enabling more efficient acquisition and orchestration of industry-specific assets to support innovation (Raisch and Krakowski, 2021; Krakowski et al., 2023). In this sense, AI functions as a critical enabler of competitive advantage and innovation performance in the digital era.

Building on this logic, advances in deep learning, generative algorithms, and data analytics empower enterprises to create novel technologies and products, facilitating breakthrough innovation that not only propels industry evolution but also shapes consumer demand (Mariani and Dwivedi, 2024; Roberts and Candi, 2024). However, the performance impact of AI is contingent on organizational and cognitive conditions. Not-Invented-Here Syndrome (NIHS)—a bias favoring internally developed solutions over external resources and knowledge (Hannen et al., 2019)—constitutes a salient boundary condition. NIHS can impede an enterprise’s ability to absorb and leverage external inputs, including AI technologies, data assets, and ecosystem partnerships, thereby constraining the potential of AI-driven innovation (Han et al., 2025). Accordingly, the transformative effects of AI are likely moderated by the degree to which NIHS is present within the organization.

Guided by RBV and incorporating recent developments in AI research (Krakowski et al., 2023), this study advances an integrated model in which AI use enhances innovation performance via the mediating role of breakthrough innovation, with NIHS exerting a nonlinear moderating influence on this relationship. This theorization enables a nuanced account of the mechanisms and boundary conditions underlying AI-driven innovation, offering actionable implications for sustaining innovation performance in highly competitive environments.

### 2.2 Artificial intelligence usage and innovation performance

The use of artificial intelligence (AI) refers to the deployment of technologies such as machine learning, natural language processing, and computer vision by firms across domains of daily management, production, and marketing to build systems capable of simulating human intelligence (Krakowski et al., 2023). AI utilization enables firms to acquire novel resources and support innovation processes through large models, thereby enhancing innovation performance.

First, AI—by virtue of its powerful machine learning and big data analytics—can efficiently extract highly valuable innovation ideas, problem-solving strategies, industry trends, and cutting-edge technologies from vast volumes of information. It then transforms these insights into firm-specific assets, enriching the enterprise’s resource base for innovation (Ciarli et al., 2021). When such novel resources exhibit high potential value, AI further enhances firm-level innovation by integrating internal and external knowledge through its resource orchestration, thereby promoting superior innovation outcomes.

Second, AI facilitates the rapid commercialization of innovation outputs and continuously optimizes innovation strategies through real-time feedback mechanisms. By constructing interactive feedback channels among firms, markets, and end-users (Panico and Cennamo, 2022), AI enables organizations to dynamically capture user experiences and market reactions to new technologies and products through advanced data modeling. This allows firms to flexibly refine their innovation trajectories, accelerate iterative cycles of technological and product development, and ultimately secure first-mover advantages and innovation returns (Wamba-Taguimdje et al., 2020).

Third, AI empowers firms to build collaborative innovation platforms by dismantling resource and information silos, thereby facilitating cross-functional and inter-organizational resource recombination and renewal. Through intelligent matching and real-time coordination functionalities, AI enables efficient connections across intra- and inter-firm knowledge domains, forming dynamic innovation networks that enhance the circulation and sharing of knowledge and resources (Haefner et al., 2021). These networks not only accelerate innovation processes but also augment firms’ dynamic advantages to continuously sense and respond to market changes, thereby improving innovation performance. Based on the above theoretical rationale, we propose the following hypothesis:

*Hypothesis 1 (H1)*: AI usage has a positive effect on innovation performance.

### 2.3 The mediating role of breakthrough innovation

Breakthrough innovation refers to the development of technologies, products, or services that are unprecedented in the market and that catalyze significant structural shifts in existing industries (Capponi et al., 2022). It enables firms to pioneer radically new offerings, attain first-mover advantages, and reshape competitive landscapes—thereby fostering superior innovation performance and sustainable competitive advantage. The mechanisms through which AI facilitates breakthrough innovation can be delineated as follows:

First, AI—leveraging advanced algorithmic architectures and machine learning techniques—enables firms to mine core internal data across operational, managerial, and R&D domains, while simultaneously capturing critical external insights regarding market dynamics, user preferences, and frontier technologies (Raisch and Krakowski, 2021; Krakowski et al., 2023). Through predictive analytics and generative modeling, AI transforms these multi-sourced datasets into forward-looking insights, accelerating the emergence of disruptive technologies and breakthrough innovations (Mariani and Dwivedi, 2024; Roberts and Candi, 2024).

Second, AI transcends the cognitive constraints of human reasoning by exploring novel solution spaces and unconventional innovation trajectories. In virtual environments, AI enables rapid experimentation and validation of innovative ideas, facilitating the transition from concept to prototype with reduced cost and risk. This capacity substantially enhances firms’ ability to engage in technological exploration and to deviate from dominant design logics, which are essential for generating breakthrough innovation (Haefner et al., 2021; Datta and Srivastava, 2023).

Third, AI fosters cross-boundary integration of diverse resources and knowledge domains, thus driving interdisciplinary convergence and the emergence of new business models and technological applications. AI-powered intelligent platforms dismantle traditional industry boundaries by enabling the combinatorial synthesis of heterogeneous knowledge and technologies from disparate sectors, generating novel innovation pathways and unconventional solutions (Felten et al., 2021). This form of convergence not only expands the scope of innovation but also serves as a robust engine for breakthrough innovation. Accordingly, we propose the following hypothesis:

*Hypothesis 2 (H2)*: AI usage has a positive effect on firms’ breakthrough innovation.

Breakthrough innovation plays a pivotal role in enabling firms to achieve heterogeneous leaps in highly uncertain environments and can influence innovation performance through several critical mechanisms.

First, breakthrough innovation often entails fundamental shifts in technological paradigms and product architectures, requiring firms to transcend path dependence and establish highly heterogeneous resource and knowledge configurations (Srivastava and Gnyawali, 2011). This process involves the recombination of external technologies, knowledge, and ecosystem partnerships (Dong et al., 2017), facilitating unique complementarities and synergies among resources, thereby enhancing the distinctiveness and inimitability of innovation outputs—and in turn, boosting innovation performance.

Second, as a core driver of upgrading, breakthrough innovation activates internal exploratory momentum and reinforces firms’ strategic flexibility and core competitiveness. By stimulating the development of new technological resources, it strengthens firms’ absorptive, transformative, and exploitative capacities toward novel knowledge. This dynamic enhances resource conversion efficiency from R&D to commercialization and establishes robust imitation barriers, supporting sustainable competitive advantage rooted in technological leadership and organizational responsiveness (Silva et al., 2017).

Third, breakthrough innovation opens up new avenues for sustained growth by enabling firms to transcend existing industry boundaries and redefine value creation logics and competitive rules (Markides, 2006). Through the launch of highly differentiated and disruptive offerings, firms can rapidly enter emerging markets, secure first-mover dominance, and establish new industry standards or consumer paradigms (Bennett and Hauser, 2013). These strategic gains contribute to stronger entry barriers and customer lock-in mechanisms, thereby reinforcing long-term improvements in innovation performance. Based on the above reasoning, we propose the following hypothesis:

*Hypothesis 3 (H3)*: Breakthrough innovation has a positive effect on innovation performance.

Artificial intelligence (AI), as a general-purpose enabling technology, is profoundly reshaping the logic of innovation and the boundaries of organizational resources (Mariani and Dwivedi, 2024). Existing research suggests that the extensive deployment of AI not only enhances firms’ operational efficiency and information processing capacity but also energizes breakthrough innovation by augmenting organizational cognition, reconfiguring innovation trajectories, and fostering cross-domain integration (Datta and Srivastava, 2023; Krakowski et al., 2023). Simultaneously, breakthrough innovation itself—as a form of strategic innovation—substantially strengthens firms’ heterogeneous competitive advantage and contributes to sustained innovation performance (Capponi et al., 2022). Building on these insights, this study posits that breakthrough innovation mediates the relationship between AI usage and innovation performance, for the following reasons:

First, by leveraging natural language processing, image recognition, and deep learning algorithms, AI empowers firms to efficiently capture signals related to technological evolution, latent customer needs, and untapped market domains (Roberts and Candi, 2024). This cognitive augmentation expands their technological imagination, while simultaneously enhancing their sensitivity to market opportunities (Haefner et al., 2021). As a result, firms can enact fundamental shifts in technological paradigms and product concepts, generating breakthrough innovations characterized by first-mover potential and disruptive impact. Given their high degree of differentiation and technological inimitability, such innovations often yield rapid returns in both output and market performance, thereby improving overall innovation performance (Srivastava and Gnyawali, 2011).

Second, AI-enabled virtual simulation, intelligent optimization, and generative modeling technologies offer firms agile and cost-efficient platforms for experimentation (Datta and Srivastava, 2023). This allows firms to validate the feasibility and market response of new products or technological pathways at early conceptual stages, significantly reducing the trial-and-error costs and path dependencies typically associated with breakthrough innovation (Silva et al., 2017). More importantly, the iterative optimization mechanisms embedded in AI systems facilitate the seamless transformation of breakthrough innovation outputs from conceptual prototypes to market-ready applications, thereby contributing to enhanced innovation performance.

Third, AI-driven intelligent platforms enable the fluid circulation and recombination of data, resources, and knowledge across industrial, organizational, and geographic boundaries (Felten et al., 2021). By dismantling traditional structural barriers, AI allows firms to access and recombine heterogeneous knowledge domains and resources, thereby unlocking the potential for cross-disciplinary innovation. In this context, breakthrough innovation transcends the evolution of individual product lines or core technologies, instead taking the form of systemic transformation across technologies and industries (Markides, 2006). This mechanism opens new market spaces and facilitates the redefinition of industry rules, providing firms with scalable pathways for value creation and long-term innovation returns. Based on the above reasoning, we propose the following hypothesis:

*Hypothesis 4 (H4)*: Breakthrough innovation mediates the relationship between AI usage and innovation performance.

### 2.4 The moderating role of NIHS

Not-Invented-Here Syndrome (NIHS) denotes an enterprise- and manager-level propensity to privilege internally developed solutions while discounting or resisting external technologies and products, thereby exhibiting reluctance to adopt or leverage them (Ismail et al., 2023). NIHS signals limited openness to collaboration and an overreliance on in-house R&D, which can impede interorganizational resource and technology exchange and, in turn, constrain innovation capacity. Extant research examines NIHS through psychological and organizational learning lenses. Psychologically, NIHS reflects a preference for internal knowledge and technology that can strengthen resource integration and support independent innovation—especially in technology-intensive, patent-protected settings where knowledge appropriation and secrecy are paramount (Antons and Piller, 2015; Han et al., 2025). Conversely, NIHS can also foster cognitive rigidity and a closed interpretive frame, prompting systematic rejection or neglect of external knowledge and emerging technologies. Such closure undermines the acquisition, absorption, and application of diverse information in dynamic environments (Hannen et al., 2019; Ismail et al., 2023), narrows open innovation channels, and diminishes the likelihood of breakthrough outcomes, thereby depressing overall innovation performance. Accordingly, the effects of NIHS are unlikely to be linear. Rather, they are contingent and potentially nonlinear—shaped by underlying psychological mechanisms and organizational learning processes, and varying with technological complexity, appropriability conditions, and environmental dynamism. This perspective motivates theorizing NIHS as a multidimensional, context-dependent construct with curvilinear implications for enterprise innovation.

Building on this analysis, we argue that the effect of AI usage on innovation performance is likely moderated by NIHS in a nonlinear manner. Specifically, at low levels of NIHS, firms are highly open to external technologies and resources, actively acquiring external knowledge to support innovation (Dong and Netten, 2017; Amann et al., 2021). However, such openness may lead to information overload and resource redundancy. In this scenario, AI becomes overburdened by excessive data processing and fails to effectively integrate and optimize key resources, weakening its ability to enhance innovation through precise decision support, product innovation, and faster market responsiveness. Therefore, low levels of NIHS may attenuate the positive impact of AI on innovation performance due to resource fragmentation and overload.

At moderate levels of NIHS, firms achieve an optimal balance between openness and closure. They establish selective barriers, effectively absorbing high-quality external resources while avoiding redundancy and overload (Wu et al., 2022). In this “golden zone” of resource allocation, AI can efficiently process user needs, market trends, and technological developments, improving resource orchestration and empowering firms to swiftly adjust strategies and identify latent innovation opportunities, thereby boosting innovation performance (Krakowski et al., 2023).

At high levels of NIHS, firms’ rejection of external resources intensifies. They mainly rely on internal resources and innovation pathways, with limited absorption of external technologies or market insights (Hannen et al., 2019; Han et al., 2025). This inward focus reduces firms’ sensitivity to market trends and emerging technologies, constraining the role of AI in the innovation process. AI is then limited to optimizing internal resources, failing to exploit novel external knowledge, which ultimately undermines innovation performance (Shan et al., 2020; Broekhuizen et al., 2023). Based on this reasoning, we propose the following hypothesis:

*Hypothesis 5 (H5)*: NIHS has a nonlinear moderating effect on the relationship between AI usage and innovation performance, with this relationship being strongest at moderate levels of NIHS and weaker at low or high levels.

Regarding the relationship between AI usage and breakthrough innovation, low NIHS encourages firms to acquire a large volume of external technologies and resources (Hannen et al., 2019). However, this can also introduce resource redundancy and quality heterogeneity, leading to information overload and disorganization. Such resource fragmentation weakens the ability of AI to intelligently analyze market gaps, foster innovation, and overcome bottlenecks, thereby limiting the potential for breakthrough innovation (Srivastava and Gnyawali, 2011).

At moderate levels of NIHS, firms can flexibly select, search, and integrate valuable external resources while avoiding the inefficiencies associated with excessive openness (Thornton et al., 2019; Ganco et al., 2020). This balance enables firms to fully leverage AI’s potential to intelligently identify market needs, innovation opportunities, and frontier technologies, rapidly validate new products in the marketplace, and gain timely feedback (Sullivan and Wamba, 2024). As a result, firms can retain breakthrough innovation outputs with greater market viability. Thus, a moderate level of NIHS creates the most favorable environment for AI to support breakthrough innovation.

When NIHS is excessively high, firms adopt innovation strategies that almost entirely reject external resources (Antons et al., 2017), severely limiting their potential to exploit external knowledge. In this situation, AI’s ability to predict market trends, overcome technological bottlenecks, and explore technological frontiers is significantly weakened. Firms may fail to capture emerging market opportunities or disruptive technological shifts, thereby missing crucial breakthrough innovation initiatives (Antons and Piller, 2015; Mariani and Dwivedi, 2024). Consequently, high NIHS severely undermines the potential of AI to support breakthrough innovation. Based on this reasoning, we propose the following hypothesis:

*Hypothesis 6 (H6)*: NIHS has a nonlinear moderating effect on the relationship between AI usage and breakthrough innovation, with this relationship being strongest at moderate levels of NIHS and weaker at low or high levels.

With respect to the relationship between breakthrough innovation and innovation performance, we argue that at low levels of NIHS, firms can access industry development trends, emerging opportunities, and competitor dynamics, thereby enriching their resource base (Hannen et al., 2019). While low-quality resources and increased managerial complexity may exist, breakthrough innovation can effectively mitigate risks and uncertainties in the innovation process, leveraging available industry resources to support the commercialization of breakthrough products and technologies, thus generating high innovation returns (Dong et al., 2017). Therefore, although low NIHS may lead to resource redundancy and managerial challenges, breakthrough innovation remains sufficient to drive superior innovation performance.

As NIHS increases, firms’ openness to external resources declines (Antons et al., 2017), resulting in strategic dilemmas about whether to rely on external resources for technology commercialization, focus solely on internal R&D, or attempt a hybrid approach (Felin and Zenger, 2014). In such cases, even when breakthrough innovation exists, the simultaneous need to integrate internal and external resources can consume considerable time and energy, dispersing strategic focus and delaying product commercialization, which diminishes the positive effect of breakthrough innovation on innovation performance (Manzini et al., 2017).

At very high levels of NIHS, firms compensate by intensifying R&D investments, promoting internal learning, and strengthening employee training to boost internal innovation, thus reducing dependency on external resources (Zhang and Tang, 2017). This strategy not only reduces the costs of searching and absorbing external knowledge but also activates internal innovation potential (Scuotto et al., 2017). Under these conditions, breakthrough innovation generates substantial competitive advantages and bargaining power, establishes imitation barriers, and significantly improves innovation performance. Accordingly, we propose the following hypothesis:

*Hypothesis 7 (H7)*: NIHS has a nonlinear moderating effect on the relationship between breakthrough innovation and innovation performance, with this relationship being strongest at low and high levels of NIHS and weaker at moderate levels.

**Figure 1:**
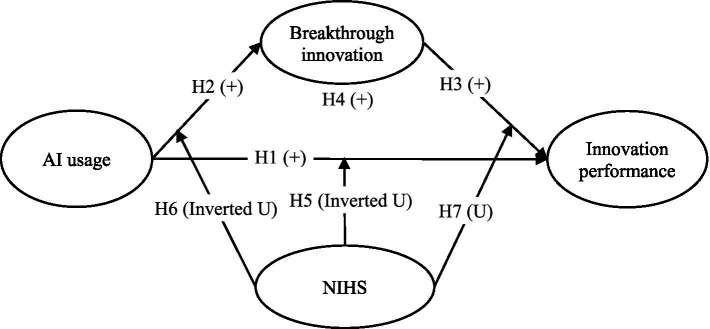
Theoretical model.

Based on these arguments, we construct the research model as shown in Figure 1.

## 3 Methodology

### 3.1 Sample and data collection

This study examines whether enterprises’ use of artificial intelligence (AI) enhances innovation performance by enabling breakthrough innovation, with a particular focus on the nonlinear moderating role of Not-Invented-Here Syndrome (NIHS). We concentrate on high-tech enterprises to ensure representativeness and external validity. These enterprises possess strong technological foundations and sustained innovation incentives and are among the earliest and most intensive adopters of AI in R&D and managerial processes (Raisch and Krakowski, 2021). High-tech enterprises also frequently pursue breakthrough innovation—developing disruptive technologies and probing emerging markets—making them an apt context to observe AI-enabled resource recombination, knowledge discovery, and shifts in innovation trajectories. Following, we define high-tech enterprises as organizations led by technical professionals, grounded in the commercialization of scientific and technological outputs, and engaged in sustained technological innovation that contributes to economic performance. Typical attributes include high R&D intensity, knowledge concentration, rapid technological renewal, and substantial innovation investment, with broad coverage across biopharmaceuticals, AI, information technology, new materials, and renewable energy (Han et al., 2024). This context supports the collection of extensive, high-quality data for the present research. For methodological transparency, we designed a structured questionnaire with four sections. The first communicates the study purpose, confidentiality assurances, and informed-consent procedures, ensuring fully informed participation. The second measures the focal constructs—AI usage, breakthrough innovation, innovation performance, and perceived NIHS—using 7-point Likert scales. All items are adapted from established instruments and calibrated to the high-tech enterprise context to maximize validity and relevance. The third section captures respondent demographics (e.g., gender, age, education, managerial role, functional area) and enterprise-level attributes (e.g., founding year, size, business scope, core technology domain), both to verify the high-tech profile and to serve as controls in subsequent analyses. The fourth comprises open-ended questions eliciting qualitative insights on AI implementation pathways, innovation strategies, future technological trajectories, and mechanisms for integrating external resources (e.g., research institutes, open platforms, strategic partners). This section also functions as a validity check by assessing respondents’ professional understanding, thereby supporting the authenticity and reliability of responses—an especially critical consideration when probing the cognitive depth of technology-intensive enterprises.

To obtain sectorally broad and geographically diverse evidence while retaining high-tech characteristics, we administered a two-wave longitudinal global online survey via Prolific, a widely used platform for high-quality management research (Jeesha and Purani, 2021; Kossyva et al., 2023; Han et al., 2025). Prolific’s verified respondent profiles, quality-control protocols, and support for rigorous sampling criteria make it well-suited to studies of complex managerial constructs. For longitudinal linkage, the platform assigns each participant a unique, system-generated, anonymous identifier upon initial entry, enabling precise matching of responses across waves without collecting or storing personally identifiable information (e.g., names, emails, IP addresses) at any stage. This mechanism preserves confidentiality and data security while ensuring reliable panel matching, thereby providing a robust basis for our two-stage data collection and analysis.

Prior to the formal survey, we conducted a two-week pilot study with 100 senior managers from high-tech firms engaged in AI application and breakthrough innovation practices. The aim was to iteratively refine questionnaire logic, language fit, and construct operationalization. During the pilot phase, we implemented several screening and validation mechanisms: (1) Enterprise qualification verification: Respondents were required to provide their company’s registered name or official website to verify that the firm is innovation-driven and technology-intensive, with strict confidentiality guaranteed; (2) Background verification: we cross-berified firm details using IP addresses, country/region codes, and public web information to confirm the existence and technological focus of the firms; (3) Cognitive competency screening: Open-ended responses were reviewed to assess whether respondents demonstrated practical understanding of AI and innovation management, ensuring the exclusion of low-validity responses; and (4) Response consistency analysis: Correlations between key variable items and open-ended responses were analyzed to identify logical inconsistencies and filter out unreliable data.

To capture the temporal dynamics of innovation and strengthen causal inference, we employed a two-wave longitudinal survey with a 12-month interval. This design serves several methodological objectives. First, temporally separating predictors (Time 1) from outcomes (Time 2) establishes causal precedence and mitigates common method bias (Podsakoff et al., 2003). Second, the one-year lag aligns with prior research and industry practice in technology-intensive settings, allowing sufficient time for AI initiatives to yield observable effects on innovation performance (Han et al., 2024). In implementation, we fielded the first wave in April 2024, distributing 600 questionnaires to middle- and senior-level managers in technology-intensive enterprises and obtaining 585 valid responses (97.5% response rate). One year later, we administered the second wave via Prolific to the same cohort, yielding 355 valid responses (60.7% retention). To ensure panel integrity and confidentiality, we used Prolific’s anonymous identifiers to match respondents across waves without collecting personally identifiable information. This sampling and linkage protocol enhances the methodological rigor and credibility of our study.

The resulting panel constitutes a valid sample for empirical analysis. Table 1 reports descriptive statistics at both the individual and enterprise levels. Individually, 59.72% of respondents were male and 40.28% female. The plurality were aged 26–35 (42.82%), followed by 36–45 (26.76%), under 25 (16.90%), and 46 or above (13.52%). Regarding education, 56.90% held a bachelor’s degree, 20.85% had a junior-college degree or below, 20.28% held a master’s degree, and 1.97% possessed a doctoral or postdoctoral degree. In terms of managerial position, 46.48% were in middle management, 20.56% in senior management, 16.06% served as chairpersons or general managers, and 16.90% held other managerial roles. Functionally, most were responsible for product (31.55%), technology (20.56%), or marketing (19.44%); smaller shares worked in R&D (8.17%) or human resources (3.94%). At the enterprise level, 40.00% had operated for more than 10 years, 21.97% for 5–10 years, 19.72% for 3–5 years, and 16.90% for fewer than 3 years. Sectorally, the sample was concentrated in high-end manufacturing and intelligent hardware (21.69%), with additional representation from new materials, biopharmaceuticals, artificial intelligence and big data, information technology, and other technology-intensive fields. With respect to size, 67.32% employed fewer than 100 people, 13.80% had 101–300, 4.23% had 501–1,000, and 7.32% had 301–500 employees; a further 7.32% reported 1,001 or more employees. While the sample is diverse, it is somewhat unbalanced across several dimensions.

**Table 1:** Descriptive statistics of the sample.

Characteristics	Types	Number	Percentage
Gender	Male	212	59.72%
	Female	143	40.28%
Age	≤25 years old	60	16.90%
	26–35 years old	152	42.82%
	36–45 years old	95	26.76%
	>46 years old	48	13.52%
Education	Junior college and below	74	20.85%
	Undergraduate	202	56.90%
	Master degree	72	20.28%
	Doctoral and Postdoctoral	7	1.97%
Position	Chairman or general manager	57	16.06%
	Senior management	73	20.56%
	Middle management	165	46.48%
	Other	60	16.90%
Responsible field	Research and Development (R&D)	29	8.17%
	Technology	73	20.56%
	Product	112	31.55%
	Marketing	69	19.44%
	Human Resources (HR); Finance; Public Relation	14	3.94%
	Others	58	16.34%
Established years	<3 years	60	16.90%
	3–5 years	70	19.72%
	5–10 years	78	21.97%
	>10 years	142	40.00%
Industry	High-end Manufacturing and Intelligent Hardware	77	21.69%
	New Materials	67	18.87%
	Biopharmaceuticals	60	16.90%
	Artificial Intelligence and Big Data	58	16.34%
	Information Technology (including ICT, Software, and Platform Services)	45	12.68%
	Other Technology-intensive Sectors	48	13.52%
Number of employees	≤100	239	67.32%
	101–300	49	13.80%
	301–500	26	7.32%
	501–1,000	15	4.23%
	≥1,001	26	7.32%

### 3.2 Variable measurement

All variables in this study were measured using adapted scales based on prior validated research. A detailed list of measurement items is provided in the Appendix. Each construct was assessed using a five-point Likert scale ranging from 1 (“strongly disagree”) to 5 (“strongly agree”).

Innovation performance was measured using a five-item scale developed by existing research (Zeng et al., 2010), which evaluates the effectiveness of innovation outcomes in terms of market influence, operational efficiency, and competitive advantage.

To assess the extent of AI usage, we adopted a three-item scale that focuses on the degree to which AI technologies are integrated into organizational processes and operations (Han et al., 2025).

Breakthrough innovation refers to the firm’s ability to achieve radical advances in technologies, products, services, or business models that significantly disrupt market structures or industry dynamics (Capponi et al., 2022; Datta and Srivastava, 2023). Drawing from existing literature, we designed a four-item scale to measure a firm’s breakthrough innovation.

Not-Invented-Here Syndrome (NIHS) captures the extent to which firm leaders exhibit resistance to external knowledge or technology inputs (Amann et al., 2021). Following previously validated instruments, we employed a three-item scale adapted from existing studies (Burcharth et al., 2014; Arias-P’Erez and V’Elez-Jaramillo, 2022). For example, one item reads: “I believe external knowledge is as valuable as internally developed knowledge.”

Control Variables: To enhance the robustness of our findings, we included several control variables at both the individual and firm levels. At the individual level, we controlled for gender, age, educational background, and managerial position to mitigate potential biases arising from personal perceptions or decision-making behavior. At the firm level, we controlled for business scope, firm age, employee size, and primary industry sector.

### 3.3 Data analysis

Partial Least Squares Structural Equation Modeling (PLS-SEM) is widely used in strategy and innovation research (Hair et al., 2012; Han et al., 2022), making it an appropriate and defensible methodological choice for this study. We adopt PLS-SEM for three principal reasons. First, our objective is prediction-oriented—to identify key drivers and explain variance in the focal outcomes across multiple interrelated constructs. PLS-SEM prioritizes maximization of explained variance, aligning closely with these aims. Second, the model comprises several composite constructs—AI usage, breakthrough innovation, NIHS, and innovation performance—and entails testing both mediation and moderation. PLS-SEM is well suited to such complexity, offering a robust framework for estimating indirect and interaction effects in multifaceted structural models (Hair et al., 2012; Hashi and Stojčić, 2013; Cepeda-Carrion et al., 2019). Third, PLS-SEM performs reliably with small to large samples and under non-normal data conditions, thereby enhancing result stability and supporting rigorous hypothesis testing (Willaby et al., 2015; Hair et al., 2017). Accordingly, PLS-SEM best fits our research objectives and data characteristics and is employed for the empirical analyses.

In implementing this methodology, we employed SmartPLS 4 software to conduct path modeling and hypothesis testing. To ensure the reliability and statistical validity of our results, we employed a nonparametric bootstrapping procedure with 5,000 subsamples for estimating indirect (mediated) effects. The data analysis proceeded in two stages. First, we assessed the measurement model to evaluate construct reliability and validity, including tests of internal consistency, convergent validity, and discriminant validity. Second, we evaluated the structural model to test the hypothesized relationships between constructs (Henseler et al., 2016). This methodological approach allows for a comprehensive and systematic examination of complex inter-variable relationships, thereby ensuring the scientific rigor and credibility of the study’s conclusions.

## 4 Results and analysis

### 4.1 Common method bias

To enhance data reliability and mitigate common method bias (CMB), we implemented both procedural and statistical remedies. Procedurally, the survey was administered anonymously, and item order was randomized to attenuate priming and order effects (Palacios-Manzano et al., 2021). To assess multicollinearity, we computed variance inflation factors (VIFs)—which index the inflation of coefficient variance due to collinearity among predictors—and followed the conventional guideline that VIF > 3 indicates potential concern (Hair et al., 2019). All constructs exhibited VIFs well below this threshold, suggesting that multicollinearity is not problematic.

We further evaluated CMB using multiple approaches centered on confirmatory factor analysis (CFA). A four-factor model—constraining each item to load on its theorized latent construct—served as the baseline reflecting expected discriminant validity. A competing single-factor model—loading all items from the four focal constructs onto one common factor—assessed whether a general method factor could account for the shared variance. The four-factor specification fit the data well (χ^2^/df = 2.438, CFI = 0.938, TLI = 0.931, RMSEA = 0.048) and significantly outperformed the single-factor model (Δχ^2^ = 2330.589, Δdf = 6, *p* < 0.001), indicating that any common method factor is negligible (Podsakoff et al., 2003). As an additional check, we estimated an unmeasured latent common method factor (ULCMF) model and compared it with the original measurement model. Differences in fit were marginal (Δχ^2^/df = 0.303, ΔCFI = 0.013, ΔTLI = 0.008, ΔRMSEA = 0.004) and did not exceed established cutoffs. Convergence of evidence across these diagnostics provides strong assurance that CMB is unlikely to substantially bias our findings.

The detailed results of the common method bias tests are presented in Table 2.

**Table 2:** Common method bias analysis.

Model	χ^2^	df	χ^2^/*df*	CFI	TLI	RMSEA
Single-factor model	2986.411	275	10.86	0.743	0.720	0.137
Four-factor model	655.822	269	2.438	0.938	0.931	0.048
ULCMF	520.943	244	2.135	0.951	0.939	0.044

### 4.2 Measurement model evaluation

To ensure data quality and bolster the robustness of our findings, we conducted a comprehensive evaluation of the measurement model’s reliability and validity. First, we assessed internal consistency—the extent to which items within a scale coherently reflect the same latent construct—using Cronbach’s alpha and Dillon–Goldstein’s rho (composite reliability in the PLS literature). As reported in Table 3, both coefficients exceeded the recommended 0.70 threshold for all constructs, indicating satisfactory internal consistency and reliability.

**Table 3:** Reliability and validity of constructs.

Variables	Item	OL	T-Value	Cr. Alpha	rho_C	AVE
Artificial intelligence usage (AIU)	AIU1	0.834	40.099	0.9	0.924	0.67
	AIU2	0.792	30.543			
	AIU3	0.872	60.733			
	AIU4	0.842	46.135			
	AIU5	0.852	48.484			
	AIU6	0.709	23.264			
Breakthrough innovation (BI)	BI1	0.777	25.394	0.744	0.839	0.568
	BI2	0.833	47.299			
	BI3	0.752	24.761			
	BI4	0.638	13.997			
Not-Invented-Here Syndrome (NIHS)	NIHS1	0.656	9.153	0.661	0.752	0.531
	NIHS2	0.764	8.949			
	NIHS3	0.671	8.417			
Innovation performance(IP)	IP1	0.77	27.816	0.838	0.885	0.608
	IP2	0.812	37.878			
	IP3	0.814	38.03			
	IP4	0.787	30.947			
	IP5	0.711	19.163			

Second, we examined convergent validity—the degree to which indicators that theoretically belong to the same construct converge empirically—based on established criteria. Specifically, we required (1) indicator loadings greater than 0.50 to evidence meaningful item–construct linkage, (2) average variance extracted (AVE) above 0.50 to indicate that the construct captures more variance than measurement error, and (3) composite reliability (CR) above 0.70 to confirm adequate reliability (Henseler et al., 2016; Johani et al., 2021). The results show that all loadings exceed 0.60, all AVEs are above 0.50, and all CRs surpass 0.70, collectively supporting strong convergent validity of the constructs employed in this study.

Third, to assess discriminant validity—the extent to which each construct is empirically distinct from the others—we applied the Fornell–Larcker criterion, a widely used approach in structural equation modeling. Specifically, we compared the square root of each construct’s average variance extracted (AVE) with its correlations with all other constructs. Discriminant validity is supported when the square root of a construct’s AVE (reported on the diagonal of the correlation matrix) exceeds its highest inter-construct correlation. All constructs satisfied this requirement (Table 4), indicating that discriminant validity is adequately established in the measurement model.

**Table 4:** Discriminant validity—Fornell-Larcker criterion.

Variables	AIU	BI	NIHS	IP
AIU	0.818			
BI	0.755	0.753		
NIHS	−0.305	−0.318	0.656	
IP	0.733	0.678	−0.305	0.78

Finally, we employed the heterotrait–monotrait (HTMT) ratio as an additional robustness check. HTMT evaluates construct distinctiveness by comparing the average correlations between indicators of different constructs (heterotrait–heteromethod) to those within the same construct (monotrait–heteromethod) (Voorhees et al., 2016). For reflective constructs, values below 0.90 are generally considered acceptable evidence of discriminant validity. As shown in Table 5, all HTMT values fall well below this threshold, further confirming the discriminant validity of the constructs in this study.

**Table 5:** Discriminant validity—Heterotrait-Monotrait ratio (HTMT).

Variables	AIU	BI	NIHS	IP
AIU				
BI	0.886			
NIHS	0.43	0.502		
IP	0.842	0.846	0.443	

### 4.3 Structural model evaluation

To evaluate in-sample explanatory power, we inspected the R^2^ coefficients for the endogenous constructs (Rigdon, 2012) The R^2^ statistic (coefficient of determination) captures the proportion of variance in an endogenous variable accounted for by its predictors; benchmarks of 0.75, 0.50, and 0.25 are conventionally interpreted as substantial, moderate, and weak, respectively (Henseler et al., 2016). In our model, the R^2^ for innovation performance (IP) is 0.59 and for breakthrough innovation (BI) is 0.582—both above the 0.50 threshold—indicating relatively strong explanatory power (Shmueli and Koppius, 2011).

We also assessed out-of-sample predictive relevance using the cross-validated redundancy Q^2^ statistic obtained via the blindfolding procedure. Q^2^ values greater than zero indicate that the model exhibits predictive capability, thereby supporting the model’s external validity (Hair et al., 2019). Using a 7-point omission distance in the blindfolding procedure, the Q^2^ values for innovation performance (Q^2^_IP = 0.527) and breakthrough innovation (Q^2^_BI = 0.56) were both substantially greater than zero, indicating that the model possesses high predictive accuracy (Cepeda-Carrion et al., 2019).

### 4.4 Hypothesis testing

The structural model offers a comprehensive assessment of the hypothesized relationships; direct, mediating, and moderating effects are summarized in Table 6. Each hypothesis was explicitly tested, and the results are detailed below with indications of support.

**Table 6:** Hypothesis testing results.

Effects	Original sample	T statistics	P	f^2^	95%CI		Hp	Supported
Direct effects						VIF		
AIU → IP	0.502	8.789	0.000	0.247	[0.39, 0.613]	2.488	H1	YES
AIU → BI	0.715	23.425	0.000	1.026	[0.652, 0.772]	1.181	H2	YES
BI → IP	0.265	4.009	0.000	0.065	[0.131, 0.389]	2.573	H3	YES
Indirect effects						VAF		
AIU → BI→IP	0.189	3.987	0.000		[0.094,0.279]	0.332	H4	YES
Moderating effects						VIF		
NIHS*AIU → IP	0.621	1.754	0.040	0.014	[−0.124, 1.259]	2.437	H5	YES
NIHS^2^*AIU → IP	−0.674	1.881	0.032	0.015	[−1.314, 0.074]			
NIHS*AIU → BI	0.261	1.173	0.241	0.006	[−0.201, 0.686]	1.101	H6	NO
NIHS^2^*AIU → BI	−0.299	1.328	0.184	0.007	[−0.719, 0.179]			
NIHS*BI → IP	−0.84	2.117	0.034	0.022	[−1.546, −0.001]	2.490	H7	YES
NIHS^2^*BI → IP	0.855	2.07	0.038	0.019	[−0.027, 1.567]			

*N* = 355; *p < 0.05, **p < 0.01, ***p < 0.001.

First, AI usage enhances enterprises’ capacity to integrate internal and external resources, foster interorganizational collaboration, and adapt to competitive dynamics. The path from AI usage to innovation performance is positive and significant (β = 0.502, t = 8.789, *p* < 0.001), supporting H1.

Second, AI functions as a powerful enabler of innovation by facilitating new resource discovery, identifying emergent market and technological trends, and accelerating the conversion of ideas into market-ready outcomes. Empirically, AI usage significantly promotes breakthrough innovation (β = 0.715, t = 23.425, *p* < 0.001), supporting H2.

Third, breakthrough innovation strengthens market leadership, expands market share, enhances core competitiveness, and raises imitation barriers, thereby improving innovation performance. The path from breakthrough innovation to innovation performance is positive and significant (β = 0.265, t = 4.009, *p* < 0.001), supporting H3.

To test mediation, we conducted a bootstrapping procedure with 5,000 subsamples. The indirect effect of AI usage on innovation performance via breakthrough innovation is significant (β = 0.189, t = 3.987, p < 0.001), supporting H4.

Regarding moderation, we examined the hypothesized inverted U-shaped moderating role of NIHS in the AI usage → innovation performance relationship. Results show a significant linear interaction (β = 0.621, t = 1.754, *p* < 0.05) and a significant quadratic interaction (*β* = −0.674, t = 1.881, p < 0.05), confirming the inverted U-shaped moderation: the positive effect of AI usage on innovation performance is strongest at moderate levels of NIHS and weaker when NIHS is low or high, supporting H5.

H6 posits an inverted U-shaped moderating effect of NIHS on the AI usage → breakthrough innovation relationship. The linear interaction is positive but not significant (β = 0.261, t = 1.173, *p* > 0.05), and the quadratic term is also not significant (β = −0.299, t = 1.328, p > 0.05). Thus, neither linear nor nonlinear moderation is supported, and H6 is not supported.

Finally, we find evidence of a U-shaped moderating effect of NIHS on the breakthrough innovation → innovation performance link: the positive association is weakest at moderate NIHS and stronger when NIHS is low or high. The linear interaction is negative and significant (β = −0.840, t = 2.117, *p* < 0.05), while the quadratic interaction is positive and significant (β = 0.955, t = 2.070, p < 0.05), supporting H7.

## 5 Discussion

This study investigates the mediating mechanisms and boundary conditions through which artificial intelligence (AI) usage influences innovation performance. The findings reveal several key insights. First, AI usage significantly enhances innovation performance, and this relationship is partially mediated by breakthrough innovation. Furthermore, the nonlinear moderating effects of the Not-Invented-Here Syndrome (NIHS) are explored. Specifically, NIHS exerts an inverted U-shaped moderation between AI usage and innovation performance, whereas a U-shaped moderation is observed in the relationship between breakthrough innovation and innovation performance.

With regard to the influence of AI usage on innovation performance, the central mechanism lies in maximizing the strategic value of AI within innovation processes. A moderate level of NIHS facilitates the flexible absorption of external resources while enabling firms to effectively integrate and optimize internal resources. This configuration enhances AI’s capacity to orchestrate internal–external resource recombination and thereby maximizes its innovation-enabling potential (Hannen et al., 2019; Ismail et al., 2023). In contrast, either excessively low or high levels of NIHS may result in overly open or overly closed resource strategies, impairing the effective deployment of AI, increasing innovation-related costs, and diminishing innovation outcomes (Felin and Zenger, 2014).

In terms of how breakthrough innovation enhances innovation performance, the critical issue concerns the firm’s ability to effectively translate disruptive ideas into strategic advantage. A moderate level of NIHS may hinder this process by forcing firms into a dilemma between exploring external resources and coordinating internal assets, thereby impeding their capacity to rapidly execute breakthrough innovations and capture market share. Conversely, low or high NIHS levels often compel firms to adopt either more open or more closed innovation strategies (Almirall and Casadesus-Masanell, 2010; Felin and Zenger, 2014), which, in turn, facilitate the commercialization of breakthrough technologies and accelerate innovation performance gains.

The hypothesized inverted U-shaped moderating effect of NIHS on the AI usage → breakthrough innovation relationship was not supported. Several considerations help explain this null result. Theoretically, the compelling, disruptive character of AI-enabled breakthrough innovation may overwhelm the constraining influence of NIHS. Whereas incremental innovation is often more sensitive to internal resistance toward external knowledge, breakthrough innovation catalyzed by advanced AI appears to be driven primarily by the technology’s transformative value—consistent with the strong direct effect of AI usage on breakthrough innovation observed in our model (Arias-P’Erez and V’Elez-Jaramillo, 2022; Li and Zhang, 2025; Sun et al., 2025). Methodologically, features of our high-tech enterprise sample may also contribute. Such enterprises tend to be relatively skilled at orchestrating internal and external resources and knowledge (Sullivan et al., 2021), which could compress the variance of NIHS and attenuate statistical power to detect interaction effects. More broadly, our findings position NIHS as a boundary condition whose moderating salience may diminish in contexts dominated by highly disruptive technologies like AI. Practically, this suggests that prioritizing technological advancement and capability building may be more consequential for achieving breakthrough innovation than efforts focused solely on mitigating internal resistance.

### 5.1 Theoretical contributions

This study develops a cohesive account of how AI usage enhances innovation performance via breakthrough innovation and how NIHS conditions these effects, yielding the following theoretical contributions:

First, this study advances understanding of how AI enhances innovation performance by catalyzing breakthrough innovation, thereby enriching research on AI’s role in innovation management. Prior work has emphasized AI’s dual potential—expanding access to innovation-relevant resources (Rammer et al., 2022; Roberts and Candi, 2024) and reshaping organizational cognition and decision processes (Du and Xie, 2021; Grewal et al., 2021)—yet whether AI use translates into sustained gains in innovation performance remains underexamined. Research explicitly theorizing and testing the mediating role of breakthrough innovation in the AI–performance nexus is particularly sparse. Given AI’s generative and emergent properties—and its synergy with advanced technologies such as large language models and quantum computing (Mariani and Dwivedi, 2024; Roberts and Candi, 2024)—systematic inquiry is warranted. By confirming AI’s positive effect on innovation performance and, critically, validating the mediating role of breakthrough innovation, this study fills a key gap and offers a more granular account of the mechanisms through which AI shapes innovation outcomes (Wamba-Taguimdje et al., 2020; Haefner et al., 2021; Bahoo et al., 2023).

Second, the study deepens the theorization of Not-Invented-Here Syndrome (NIHS) by elucidating its nonlinear moderating effects in digitally mediated innovation. Integrating psychological and organizational learning perspectives (Antons and Piller, 2015; Hannen et al., 2019), we move beyond treating NIHS solely as a constraint on external knowledge absorption (Arias-P’Erez and V’Elez-Jaramillo, 2022). Our evidence reveals dual boundary-spanning roles: an inverted U-shaped moderation of the AI usage–innovation performance link and a U-shaped moderation of the breakthrough innovation–performance link. These results challenge the prevailing view of NIHS as uniformly detrimental and extend its relevance to digital transformation contexts (Antons and Piller, 2015; Ismail et al., 2023). By highlighting NIHS’s curvilinear and context-contingent nature, we identify it as a critical cognitive boundary condition in AI-enabled innovation pathways, offering a novel lens on how cognitive biases and organizational learning dynamics jointly shape digital innovation performance (Mariani and Dwivedi, 2024; Sullivan and Wamba, 2024).

Third, the study contributes to the resource-based view (RBV) by extending its applicability to the digital era through the conceptualization of AI as a core strategic resource. While RBV posits that sustained advantage stems from resources that are valuable, rare, inimitable, and non-substitutable (Barney, 1991, 2001), we show that AI—as a digital, dynamic resource class—meets these criteria in distinctive ways. As digital technologies proliferate, AI reconfigures how enterprises assemble and deploy innovation assets (Krakowski et al., 2023; Mariani et al., 2023b). Our empirical results demonstrate that AI not only enables breakthrough innovation and elevates innovation performance but also operates within boundary conditions shaped by NIHS. By integrating AI into the RBV framework and validating its effects, we enhance RBV’s explanatory power in digital contexts and provide a renewed theoretical foundation for examining innovation strategies centered on the mobilization of digital resources (Barney et al., 2021; Helfat et al., 2023).

### 5.2 Practical implications

Building on our theoretical and empirical results, we distill the following practice-oriented managerial implications for technology-intensive enterprises:

First, institutionalize AI as a core strategic resource tailored to high-tech contexts. Medium- to large-sized technology-intensive enterprises—in high-end manufacturing, intelligent hardware, new materials, biopharmaceuticals, artificial intelligence, big data, and information technology—should embed AI as a foundational driver of innovation performance. C-suite leaders (e.g., CEO, CTO, Chief Innovation Officer) ought to spearhead enterprise-wide AI roadmaps aligned with innovation objectives, supported by cross-functional AI task forces spanning R&D, IT, product, and business units to ensure tight integration with the innovation agenda. Firms should implement phased investments in data infrastructure (e.g., cloud data platforms, scalable computing) while rolling out AI literacy programs for senior and middle managers to enable organization-wide, data-driven decision making. Clear KPI systems should track AI’s contribution to innovation (e.g., reduced time-to-market, improved R&D efficiency).

Second, deploy AI as an engine for breakthrough innovation with role clarity across R&D, product, and business development. R&D and Technology leaders should leverage AI-enabled discovery platforms that fuse internal R&D data with external publications and patent databases to surface transformative opportunities. Product and Engineering managers can establish “AI sandboxes” to experiment with radical concepts under controlled risk, and use AI-driven incubators to identify promising technology combinations and adjacent markets. Strategic Planning and Business Development should build partnership frameworks with research institutions and use AI to continuously scan and evaluate emergent technologies, maintaining access to frontier knowledge and capabilities.

Third, manage NIHS dynamically in line with the innovation strategy, with explicit accountabilities for HR, knowledge management, and innovation teams. Organizations should routinely assess Not-Invented-Here Syndrome (NIHS) via employee surveys and collaboration-pattern analytics coordinated by HR and knowledge management. For incremental innovation strategies, sustaining moderate NIHS—balancing external collaboration with internal capability building—is advisable. For breakthrough strategies, leaders should choose between cultivating low-NIHS environments through open-innovation platforms or intentionally maintaining high-NIHS “skunkworks” units to protect proprietary development. Knowledge systems should classify and appraise external technologies by integration complexity and strategic fit to optimize portfolio-level innovation impact.

### 5.3 Limitations and future research

Despite offering valuable insights into the mechanisms linking AI usage to innovation performance, this study has several limitations that warrant future exploration. First, different industries are subject to varying market dynamics and technological contexts, and firm size or development stage may influence how AI deployment affects innovation outcomes. Future research could conduct comparative analyses across industries, firm sizes, and life cycle stages to uncover the contextual contingencies and boundary conditions shaping the AI–innovation relationship. Such studies would provide more tailored and practical guidance for different types of enterprises.

Second, the current study primarily adopts a firm-level perspective and does not account for individual-level factors such as managerial traits or team dynamics. Future research may delve deeper into micro-foundations by examining how characteristics like decision-making style, leadership orientation, and technological adaptability of managers influence AI utilization and innovation performance. This direction would enrich theoretical frameworks on AI-enabled innovation and offer more actionable insights for organizational implementation.

## 6 Conclusion

With the rapid advancement of digital technologies such as large language models and quantum computing, AI has emerged as a pivotal strategic resource for enhancing innovation performance and firm competitiveness. However, scholarly consensus on how AI contributes to innovation remains limited, particularly concerning the mediating mechanisms and boundary conditions involved. Grounded in the resource-based view, this study develops a theoretical framework to examine the effect of AI usage on innovation performance, emphasizing the mediating role of breakthrough innovation and the moderating role of the Not-Invented-Here Syndrome (NIHS). Empirical analysis using structural equation modeling confirms that AI usage positively affects innovation performance and that breakthrough innovation serves as a significant mediator in this relationship. Furthermore, NIHS demonstrates an inverted U-shaped moderating effect between AI usage and innovation performance, and a U-shaped moderating effect between breakthrough innovation and innovation performance. These findings reinforce the role of AI as a strategic enabler of innovation and highlight the critical intermediary function of breakthrough innovation. Moreover, this study underscores the importance of achieving an optimal cognitive balance between openness and defensiveness—captured by NIHS—in the AI-driven innovation process. By doing so, it extends the applicability of the resource-based view in digital contexts and offers actionable implications for firms seeking to harness AI for sustained innovation performance and competitive advantage.

## Data Availability

The raw data supporting the conclusions of this article will be made available by the authors, without undue reservation.

## Appendix

### Innovation performance (IP)

IP1: Compared with competitors in the industry, our company has a higher number of product or service innovations.

IP2: Compared with competitors in the industry, our company achieves higher profitability from our new products or services.

IP3: Compared with competitors in the industry, our company generates higher sales from our new products or services.

IP4: Compared with competitors in the industry, our company has implemented a higher number of business process innovations.

IP5: Compared with competitors in the industry, our company has gained greater flexibility owing to improved operational processes.

### Artificial intelligence usage (AIU)

AIU1: Our company used artificial intelligence to carry out most of our job functions.

AIU2: Our company spent most of the time working with artificial intelligence.

AIU3: Our company worked with artificial intelligence in making major work decisions.

AIU4: Our company employs the most advanced AI technology.

AIU5: Our company is always the first to adopt new AI technologies in the industry.

AIU6: Our company is regarded as a leader in the latest AI technologies for film in the industry.

### Breakthrough innovation (BI)

BI1: Our company excels at developing entirely new film products that redefine the market.

BI2: Our company is at the forefront of developing groundbreaking technologies for the film industry.

BI3: Our company successfully achieves mass production of new technologies in film products.

BI4: Our company proactively adopts entirely new equipment and tools to revolutionize film project processes.

### Not-Invented-Here Syndrome (NIHS)

NIHS1: Our company is skeptical about integrating external technology for film production.

NIHS2: Our company embraces the adoption of external technologies, particularly those that align closely with our existing capabilities.

NIHS3: Our company frequently experiments with cutting-edge technologies beyond our core expertise in film production.

## References

[ref1] Almirall E. Casadesus-Masanell R. (2010). Open Versus Closed Innovation: A Model of Discovery and Divergence. Acad. Manag. Rev. 35, 27–47. doi: 10.5465/AMR.2010.45577790

[ref2] Amann M. Granstrom G. Frishammar J. Elfsberg J. (2021). Mitigating not-invented-here and not-sold-here problems: The role of corporate innovation hubs. Technovation 102377. doi: 10.1016/j.technovation.2021.102377

[ref3] Antons D. Declerck M. Diener K. Koch I. Piller F. T. (2017). Assessing the not-invented-here syndrome: Development and validation of implicit and explicit measurements. J. Organ. Behav. 38, 1227–1245. doi: 10.1002/job.2199

[ref4] Antons D. Piller F. T. (2015). Opening the black box of "not invented here": Attitudes, decision biases, and behavioral consequences. Acad. Manag. Perspect. 29, 193–217. doi: 10.5465/amp.2013.0091

[ref5] Arias-P’Erez J. E. V’Elez-Jaramillo J. (2022). Ignoring the three-way interaction of digital orientation, Not-invented-here syndrome and employee's artificial intelligence awareness in digital innovation performance: A recipe for failure. Technol. Forecasting Soc. Change 174:121305. doi: 10.1016/j.techfore.2021.121305

[ref6] Bahoo S. Cucculelli M. Qamar D. (2023). Artificial intelligence and corporate innovation: A review and research agenda. Technol. Forecast. Soc. Chang. 188:122264. doi: 10.1016/j.techfore.2022.122264

[ref7] Balasubramanian N. Ye Y. Xu M. (2022). Substituting human decision-making with machine learning: Implications for organizational learning. Acad. Manag. Rev. 47, 448–465. doi: 10.5465/amr.2019.0470

[ref8] Barney J. B. (2001). Resource-based theories of competitive advantage: A ten-year retrospective on the resource-based view. J. Manag. 27, 643–650. doi: 10.1177/014920630102700602

[ref9] Barney J. B. (1991). Firm resources and sustained competitive advantage. J. Manag. 17, 3–10. doi: 10.1016/S0742-3322(00)17018-4

[ref10] Barney J. B. Ketchen D. J. Wright M. (2021). Resource-based theory and the value creation framework. J. Manag. 47, 1936–1955. doi: 10.1177/01492063211021655

[ref11] Bennett C. C. Hauser K. (2013). Artificial intelligence framework for simulating clinical decision-making: A Markov decision process approach. Artif. Intell. Med. 57, 9–19. doi: 10.1016/j.artmed.2012.12.003, 23287490

[ref12] Broekhuizen T. Dekker H. Faria P. D. Firk S. Nguyen D. K. Sofka W. (2023). AI for managing open innovation: Opportunities, challenges, and a research agenda. J. Bus. Res. 167:114196. doi: 10.1016/j.jbusres.2023.114196

[ref13] Burcharth A. L. D. A. Knudsen M. P. Søndergaard H. A. (2014). Neither invented nor shared here: The impact and management of attitudes for the adoption of open innovation practices. Technovation 34, 149–161. doi: 10.1016/j.technovation.2013.11.007

[ref14] Capponi G. Martinelli A. Nuvolari A. (2022). Breakthrough innovations and where to find them. Res. Policy 51:104376. doi: 10.1016/j.respol.2021.104376

[ref15] Cepeda-Carrion G. Cegarra-Navarro J.-G. Cillo V. (2019). Tips to use partial least squares structural equation modelling (pls-sem) in knowledge management. J. Knowl. Manag. 23, 67–89. doi: 10.1108/JKM-05-2018-0322

[ref16] Ciarli T. Kenney M. Massini S. Piscitello L. (2021). Digital technologies, innovation, and skills: Emerging trajectories and challenges. Res. Policy 50:104289. doi: 10.1016/j.respol.2021.104289

[ref17] Cooper S. C. Pereira V. Vrontis D. Liu Y. (2023). Extending the resource and knowledge based view: Insights from new contexts of analysis. J. Bus. Res. 156:113523. doi: 10.1016/j.jbusres.2022.113523

[ref18] Datta A. A. Srivastava S. (2023). (Re) conceptualizing technological breakthrough innovation: A systematic review of the literature and proposed framework. Technol. Forecast. Soc. Chang. 194:122740. doi: 10.1016/j.techfore.2023.122740

[ref19] Dong J. Q. Mccarthy K. J. Schoenmakers W. W. M. E. (2017). How central is too central? Organizing interorganizational collaboration networks for breakthrough innovation. J. Prod. Innov. Manag. 34, 526–542. doi: 10.1111/jpim.12384

[ref20] Dong J. Q. Netten J. (2017). Information technology and external search in the open innovation age: New findings from Germany. Technol. Forecast. Soc. Chang. 120, 223–231. doi: 10.1016/j.techfore.2016.12.021

[ref21] Du S. Xie C. (2021). Paradoxes of artificial intelligence in consumer markets: Ethical challenges and opportunities. J. Bus. Res. 129, 961–974. doi: 10.1016/j.jbusres.2020.08.024

[ref22] Felin T. Zenger T. R. (2014). Closed or open innovation? Problem solving and the governance choice. Res. Policy 43, 914–925. doi: 10.1016/j.respol.2013.09.006

[ref23] Felten E. Raj M. Seamans R. (2021). Occupational, industry, and geographic exposure to artificial intelligence: A novel dataset and its potential uses. Strateg. Manag. J. 42, 2195–2217. doi: 10.1002/smj.3286

[ref24] Ganco M. Kapoor R. Lee G. K. (2020). From rugged landscapes to rugged ecosystems: Structure of interdependencies and firms innovative search. Acad. Manag. Rev. 45, 646–674. doi: 10.5465/amr.2017.0549

[ref25] Grewal D. Guha A. Satornino C. B. Schweiger E. B. (2021). Artificial intelligence: The light and the darkness. J. Bus. Res. 136, 229–236. doi: 10.1016/j.jbusres.2021.07.043

[ref26] Haefner N. Wincent J. Parida V. Gassmann O. (2021). Artificial intelligence and innovation management: A review, framework, and research agenda. Technol. Forecast. Soc. Chang. 162:120392. doi: 10.1016/j.techfore.2020.120392

[ref27] Haenlein M. Kaplan A. (2019). A brief history of artificial intelligence: On the past, present, and future of artificial intelligence. Calif. Manag. Rev. 61, 5–14. doi: 10.1177/0008125619864925

[ref28] Hair J. Hollingsworth C. L. Randolph A. B. Chong A. Y. L. (2017). An updated and expanded assessment of PLS-SEM in information systems research. Ind. Manag. Data Syst. 117, 442–458. doi: 10.1108/IMDS-04-2016-0130

[ref29] Hair J. F. Ringle C. M. Sarstedt M. (2012). Partial least squares: The better approach to structural equation modeling? Long Range Plan. 45, 312–319. doi: 10.1016/j.lrp.2012.09.011

[ref30] Hair J. F. Risher J. J. Sarstedt M. Ringle C. M. (2019). When to use and how to report the results of pls-sem. Eur. Bus. Rev. 31, 2–24. doi: 10.1108/EBR-11-2018-0203

[ref31] Han W. Li X. Zhu W. Lu R. Zu X. (2024). Knowledge digitization and high-tech firm performance: A moderated mediation model incorporating business model innovation and entrepreneurial orientation. Technol. Soc. 77:102536. doi: 10.1016/j.techsoc.2024.102536

[ref32] Han W. Zhou Y. Lu R. (2022). Strategic orientation, business model innovation and corporate performance-Evidence from construction industry. Front. Psychol. 13:971654. doi: 10.3389/fpsyg.2022.971654, 36337575 PMC9631319

[ref33] Han W. Zhu W. Song Z. Lu R. (2025). Innovative resources driven artificial intelligence orientation: The moderating role of environmental and executives' characteristics. Technol. Soc. 102837. doi: 10.1016/j.techsoc.2025.102837

[ref34] Hannen J. Antons D. Piller F. Salgea T. O. Coltman T. Devinney T. M. (2019). Containing the Not-Invented-Here Syndrome in external knowledge absorption and open innovation: The role of indirect countermeasures. Res. Policy 48:103822. doi: 10.1016/j.respol.2019.103822

[ref35] Hashi I. Stojčić N. (2013). The impact of innovation activities on firm performance using a multi-stage model: evidence from the community innovation survey 4. Res. Policy 42, 353–366. doi: 10.1016/j.respol.2012.09.011

[ref36] Helfat C. E. Kaul A. Jr. Barney J. B. Chatain O. Singh H. (2023). Renewing the resource-based view: New contexts, new concepts, and new methods. Strateg. Manag. J. 44, 1357–1390. doi: 10.1002/smj.3500

[ref37] Henseler J. Ringle C. M. Sarstedt M. (2016). Testing measurement invariance of composites using partial least squares. Int. Mark. Rev. 33, 405–431. doi: 10.1108/IMR-09-2014-0304

[ref38] Ismail M. Bello-Pintado A. García-Marco T. Lazzarotti V. (2023). Enhancing open innovation: Managing not invented here syndrome in collaborative projects. Technovation 128:102879. doi: 10.1016/j.technovation.2023.102879

[ref39] Jancenelle V. E. (2021). Tangible− Intangible resource composition and firm success. Technovation 108:102337. doi: 10.1016/j.technovation.2021.102337

[ref40] Jeesha K. Purani K. (2021). Webcare as a signal: exhaustive-selective webcare strategy and brand evaluation. Eur. J. Mark. 55, 1930–1953. doi: 10.1108/EJM-05-2019-0421

[ref41] Johani F. H. Shah S. A. Safian N. (2021). Validity and reliability of biopsychosocial-related measurement scales among low-income malaysian smoker. Glob. J. Public Health Med. 3, 301–314. doi: 10.37557/GJPHM.V3I1.79

[ref42] Kossyva D. Theriou G. Aggelidis V. Sarigiannidis L. (2023). Definitions and antecedents of engagement: A systematic literature review. Manag. Res. Rev. 46, 719–738. doi: 10.1108/MRR-01-2021-0043 PMC10319181 37408885

[ref43] Krakowski S. Luger J. Raisch S. (2023). Artificial intelligence and the changing sources of competitive advantage. Strateg. Manag. J. 44, 1425–1452. doi: 10.1002/smj.3387

[ref44] Li J. Zhang X. (2025). The adoption of artificial intelligence and enterprises’ breakthrough innovation: An analysis based on China’s patent citation network. Appl. Econ., 1–16. doi: 10.1080/00036846.2025.2556045

[ref45] Manzini R. Lazzarotti V. Pellegrini L. (2017). How to remain as closed as possible in the open innovation era: The case of Lindt & Sprüngli. Long Range Plan. 50, 260–281. doi: 10.1016/j.lrp.2015.12.011

[ref46] Mariani M. Dwivedi Y. K. (2024). Generative artificial intelligence in innovation management: A preview of future research developments. J. Bus. Res. 175:114542. doi: 10.1016/j.jbusres.2024.114542

[ref47] Mariani M. M. Machado I. Magrelli V. Dwivedi Y. K. (2023a). Artificial intelligence in innovation research: A systematic review, conceptual framework, and future research directions. Technovation 122:102623. doi: 10.1016/j.technovation.2022.102623

[ref48] Mariani M. M. Machado I. Nambisan S. (2023b). Types of innovation and artificial intelligence: A systematic quantitative literature review and research agenda. J. Bus. Res. 155:113364. doi: 10.1016/j.jbusres.2022.113364

[ref49] Markides C. (2006). Disruptive innovation: In need of better theory. J. Prod. Innov. Manag. 23, 19–25. doi: 10.1111/j.1540-5885.2005.00177.x

[ref50] Palacios-Manzano M. León-Gomez A. Santos-Jaén J. M. (2021). Corporate social responsibility as a vehicle for ensuring the survival of construction SMEs. The mediating role of job satisfaction and innovation. IEEE Trans. Eng. Manag. 71, 168–181. doi: 10.1109/TEM.2021.3114441

[ref51] Panico C. Cennamo C. (2022). User preferences and strategic interactions in platform ecosystems. Strateg. Manag. J. 43, 507–529. doi: 10.1002/smj.3149

[ref52] Podsakoff P. M. Mackenzie S. B. Lee J.-Y. Podsakoff N. P. (2003). Common method biases in behavioral research: a critical review of the literature and recommended remedies. J. Appl. Psychol. 88:879. doi: 10.1037/0021-9010.88.5.879, 14516251

[ref53] Raisch S. Krakowski S. (2021). Artificial intelligence and management: The automation-augmentation paradox. Acad. Manag. Rev. 46, 192–210. doi: 10.5465/amr.2018.0072

[ref54] Rammer C. Fernández G. P. Czarnitzki D. (2022). Artificial intelligence and industrial innovation: Evidence from German firm-level data. Res. Policy 51:104555. doi: 10.1016/j.respol.2022.104555

[ref55] Rigdon E. E. (2012). Rethinking partial least squares path modeling: in praise of simple method. Long Range Plan. 45, 341–358. doi: 10.1016/j.lrp.2012.09.010

[ref56] Roberts R. L. Candi M. (2024). Artificial intelligence and innovation management: Charting the evolving landscape. Technovation 136:103081. doi: 10.1016/j.technovation.2024.103081

[ref57] Scuotto V. Giudice M. D. Peruta M. R. D. Tarba S. (2017). The performance implications of leveraging internal innovation through social media networks: An empirical verification of the smart fashion industry. Technol. Forecast. Soc. Chang. 120, 184–194. doi: 10.1016/j.techfore.2017.03.021

[ref58] Shan W. Qiao T. Zhang M. (2020). Getting more resources for better performance: The effect of user-owned resources on the value of user-generated content. Technol. Forecast. Soc. Chang. 161:120318. doi: 10.1016/j.techfore.2020.120318

[ref59] Shmueli G. Koppius O. R. (2011). Predictive analytics in information systems research. MIS Q. 35, 553–572. doi: 10.2307/23042796

[ref60] Silva G. M. Styles C. Lages L. F. (2017). Breakthrough innovation in international business: The impact of tech-innovation and market-innovation on performance. Int. Bus. Rev. 26, 391–404. doi: 10.1016/j.ibusrev.2016.10.001

[ref61] Srivastava M. K. Gnyawali D. R. (2011). When do relational resources matter? Leveraging portfolio technological resources for breakthrough innovation. Acad. Manag. J. 54, 797–810. doi: 10.5465/amj.2011.64870140

[ref62] Sullivan D. M. Marvel M. R. Wolfe M. T. (2021). With a little help from my friends? How learning activities and network ties impact performance for high tech startups in incubators. Technovation 101:102209. doi: 10.1016/j.technovation.2020.102209

[ref63] Sullivan Y. Wamba S. F. (2024). Artificial intelligence and adaptive response to market changes: A strategy to enhance firm performance and innovation. J. Bus. Res. 174:114500. doi: 10.1016/j.jbusres.2024.114500

[ref64] Sun Z. Wu X. Dong Y. Lou X. (2025). How Does Artificial Intelligence Application Enable Sustainable Breakthrough Innovation? Evidence from Chinese enterprises. Sustainability 17:7787. doi: 10.3390/su17177787

[ref65] Terziovski M. (2010). Innovation practice and its performance implications in small and medium enterprises (SMEs) in the manufacturing sector: A resource-based view. Strateg. Manag. J. 31, 892–902. doi: 10.1002/smj.841

[ref66] Thornton S. C. Henneberg S. C. Leischnig A. Naudé P. (2019). It’s in the mix: How firms configure resource mobilization for new product success. J. Prod. Innov. Manag. 36, 513–531. doi: 10.1111/jpim.12489

[ref67] Voorhees C. M. Brady M. K. Calantone R. Ramirez E. (2016). Discriminant validity testing in marketing: an analysis, causes for concern, and proposed remedies. J. Acad. Mark. Sci. 44, 119–134. doi: 10.1007/s11747-015-0455-4

[ref68] Wamba-Taguimdje S.-L. Wamba S. F. Kamdjoug J. R. K. Wanko C. E. T. (2020). Influence of artificial intelligence (AI) on firm performance: the business value of AI-based transformation projects. Bus. Process. Manag. J. 26, 1893–1924. doi: 10.1108/BPMJ-10-2019-0411

[ref69] Willaby H. W. Costa D. S. J. Burns B. D. Maccann C. Roberts R. D. (2015). Testing complex models with small sample sizes: A historical overview and empirical demonstration of what partial least squares (PLS) can offer differential psychology. Personal. Individ. Differ. 84, 73–78. doi: 10.1016/j.paid.2014.09.008

[ref70] Wilms R. Winnen L. A. Lanwehr R. (2019). Top Managers' cognition facilitates organisational ambidexterity: The mediating role of cognitive processes. Eur. Manag. J. 37, 589–600. doi: 10.1016/j.emj.2019.03.006

[ref71] Witthöft J. Aydin B. Pietsch M. (2025). Not invented here, not shared here: How school leaders’ attitudes towards external knowledge affect collaborative innovation and collective teacher innovativeness in Germany. Educational Management Administration & Leadership, 1–27. doi: 10.1177/17411432251346950

[ref72] Wu L. Sun L. Chang Q. Zhang D. Qi P. (2022). How do digitalization capabilities enable open innovation in manufacturing enterprises? A multiple case study based on resource integration perspective. Technol. Forecast. Soc. Chang. 184:122019. doi: 10.1016/j.techfore.2022.122019

[ref73] Zeng S. X. Xie X. M. Tam C. M. (2010). Relationship between cooperation networks and innovation performance of SMEs. Technovation 30, 181–194. doi: 10.1016/j.technovation.2009.08.003

[ref74] Zhang G. Tang C. (2017). How could firm's internal R&D collaboration bring more innovation? Technol. Forecast. Soc. Chang. 125, 299–308. doi: 10.1016/j.techfore.2017.07.007

