# Supplementary material for: Artificial intelligence usage, breakthrough innovation, and innovation performance in high-tech enterprises: the nonlinear moderating role of Not-Invented-Here Syndrome
Source: Front Artif Intell. 2026 Jan 30;8:1699860. doi: 10.3389/frai.2025.1699860 (PMC12902834; doi:10.3389/frai.2025.1699860)
Supplement: Supplementary file 1 [file Data_Sheet_1.docx]

**Structured Questionnaire on the Artificial Intelligence Usage in High-tech Enterprises**

Dear Respondents,

We greatly appreciate your time and willingness to engage in this academic survey. The rapid development of Artificial Intelligence (AI) has introduced significant opportunities for enhancing innovation performance within the high-tech industry. This survey, conducted by the School of Economics and Management at the University of Electronic Science and Technology of China, is specifically designed for professionals working in the high-tech sector. It seeks to explore the various impact factors of using AI within high-tech enterprises on the industry and its products. We assure you that all responses will be kept confidential and used exclusively for academic research purposes.

This survey is intended for professionals working in high-tech enterprises, including but not limited to R&D engineers, product managers, technology strategists, innovation project leaders, marketers, HR specialists, and business development specialists. Please complete the questionnaire based on your company's actual situation. If you do not work in this sector or are unfamiliar with the relevant context, please refrain from participating in this survey.

We thank you once again for your valuable participation and contribution to this research. Your insights are crucial in understanding the application and impact of AI usage in the high-tech industry.

Best regards,

School of Economics and Management

University of Electronic Science and Technology of China

Please rate the following statements on a scale of 1 to 7, where 1 = “Strongly Disagree” and 7 = “Strongly Agree”.

**1. Artificial Intelligence Usage**

(1) Our company used artificial intelligence to carry out most of our job functions.

(2) Our company spent most of the time working with artificial intelligence.

(3) Our company worked with artificial intelligence in making major work decisions.

(4) Our company employs the most advanced AI technology.

(5) Our company is always the first to adopt new AI technologies in the industry.

(6) Our company is regarded as a leader in the latest AI technologies for film in the industry.

**2. Breakthrough Innovation**

(1) Our company excels at developing entirely new film products that redefine the market.

(2) Our company is at the forefront of developing groundbreaking technologies for the film industry.

(3) Our company successfully achieves mass production of new technologies in film products.

(4) Our company proactively adopts entirely new equipment and tools to revolutionize film project processes.

**3. Not-Invented-Here Syndrome**

(1) Our company is skeptical about integrating external technology for film production.

(2) Our company embraces the adoption of external technologies, particularly those that align closely with our existing capabilities.

(3) Our company frequently experiments with cutting-edge technologies beyond our core expertise in film production.

**4. Innovation Performance**

(1) Compared with competitors in the industry, our company has a higher number of product or service innovations.

(2) Compared with competitors in the industry, our company achieves higher profitability from our new products or services.

(3) Compared with competitors in the industry, our company generates higher sales from our new products or services.

(4) Compared with competitors in the industry, our company has implemented a higher number of business process innovations.

(5) Compared with competitors in the industry, our company has gained greater flexibility owing to improved operational processes.

The following information is relevant to you and your company. Please make your selection based on the actual situation.

**5. Your gender is**

(1) Male

(2) Female

**6. Your age is**

(1) 25 years old and below

(2) 26-35 years old

(3) 36-45 years old

(4) 46 years old and above

**7. Your education degree is**

(1) College or below

(2) Bachelor's degree

(3) Master's degree

(4) PhD or post-doctoral

**8. Your position in the company is**

(1) Chairman or general manager

(2) Senior management

(3) Middle management

(4) Other

**9. Which area are you currently responsible for in your company?**

(1) Research and Development (R&D)

(2) Technology

(3) Product

(4) Marketing

(5) Human Resources (HR)

(6) Finance or public relations

(7) Others

**10. How long has your company been established?**

(1) <3 years

(2) 3-5 years

(3) 5-10 years

(4) 10 years or more

**11. What industry is your company in?**

(1) High-end manufacturing and intelligent hardware

(2) New materials

(3) Biopharmaceuticals

(4) Artificial intelligence and big data

(5) Information technology (including ICT, Software, and Platform Services)

(6) Other Technology-intensive Sectors

**12. What is the number of employees in your company?**

(1) 100 employees or fewer

(2) Between 101 and 300 employees

(3) Between 301 and 500 employees

(4) Between 501 and 1,000 employees

(5) 1001 or more

The following section contains open-ended questions. Please answer each question based on your experience and situation.

**13. In what aspects of the project has your company used AI, and what specific changes has it brought?**

________________________________________________________________
**14. What strategic initiatives has your company undertaken in the usage of AI?**

_____________________________________________ __________________

**15. What is your perspective on the impact of AI on job roles and skill requirements in the high-tech industry?**

________________________________________________________________

**16. Can you share an example where AI significantly improved the quality of firm’s product?**

________________________________________________________________

**17. Please discuss your views and expectations for the future development of AI in high-tech enterprises.**

________________________________________________________________

**18. What is your perspective on the impact of AI on job roles and skill requirements in the high-tech industry?**

________________________________________________________________
